# Challenges in Using IT Systems for Collaboration in Healthcare Services

**DOI:** 10.3390/ijerph16101773

**Published:** 2019-05-20

**Authors:** Ann Svensson

**Affiliations:** School of Business, Economics and IT, University West, P.O. 461 86 Trollhättan, Sweden; ann.svensson@hv.se

**Keywords:** frail elderly people, healthcare, IT systems, communication, collaboration, coordination, teamwork

## Abstract

Frail elderly people refer to multi-diseased and vulnerable patients in need of medication and healthcare. These patients require healthcare from several different healthcare organizations, including hospital care, primary care, and municipal care services. This situation is challenging the capacity of healthcare organizations to manage inter-professional collaboration for person-centered care. This paper aims to identify challenges associated with collaboration between different healthcare organizations, related to the use of IT systems in the daily work practice. The paper was based on a qualitative study, which included three focus group interviews, each lasting for two hours. Each focus group consisted of a hospital physician, a primary care physician, a hospital nurse, a primary care nurse, a municipal home care nurse or an assistant officer, a physical or occupational therapist, and a family member representative. The interviews were analyzed with thematic analysis. Challenges identified in the study include insufficient information exchange, inconsistencies in communication, differences in the use of IT systems, and deficient coordination. The work processes that aim to promote collaboration between different healthcare organizations need to be better organized, and the use of IT systems needs to be better aligned.

## 1. Introduction

The ageing population of the western world poses a medical challenge for present and future societies [1]. An increased number of multi-diseased and vulnerable patients, referred to as frail elderly people, will require more medication and healthcare. Frail elderly people have many adverse health outcomes [2], and they require healthcare from several different healthcare organizations, including hospital care, primary care, and municipality care services. Moreover, many patients in this patient group are repeatedly readmitted to hospitals. Frail elderly people are harmed due to the lack of coordination and unclear division of responsibilities between separate healthcare organizations [3,4]. This situation is challenging the capacity of health organizations to manage inter-professional collaboration for person-centered care using different IT systems [5,6].

In recent years, there has been an increasing demand for the use of IT systems in the healthcare work practice. IT systems can support the provision of high-quality healthcare in society. While healthcare work is often non-linear, messy, and of an ad hoc nature [7,8], the use of formal and standardized IT systems in healthcare practices can assist in structuring the work. IT systems provide an infrastructure where healthcare professionals at different healthcare organizations can exchange and communicate information about patients. Thus, IT systems can contribute to improving the quality of, and access to, healthcare [9]. IT systems have the potential to facilitate healthcare and enhance the quality of life for frail elderly people [10]. Within healthcare, there is great need for sharing of information and knowledge, and the work relies heavily on human knowledge as well as routines of healthcare organizations [11]. There is also a need for IT systems to support organizational boundary spanning of information and knowledge [12]. However, a diverse and fragmented set of IT systems is used today, which cannot be used for sharing of information and knowledge in an efficient way to support activities that are performed within healthcare [13].

The challenges associated with the use of IT systems in healthcare have been debated in recent research [13,14,15]. It has been concluded that architecture causes structural complexity and that governance deals with behavioral complexity, and it has been suggested that IT silo systems should be dismantled [13,16]. Researchers and practitioners are trying to find solutions to existing challenges associated with using IT systems to share information and knowledge in healthcare. However, healthcare requires cooperation of healthcare professionals from multiple disciplines. This adds the dimension of the need of shared mental models as well as mutual respect and trust, between professionals, the patients, and their relatives [17]. Thus, we can also talk about professional silos as a challenge. Healthcare professionals need to understand each other’s roles and perspectives and improve teamwork in order to be able to share information and knowledge with the support of IT systems and to provide qualitative and person-centered healthcare. Therefore, we need to know more about the challenges in healthcare related to collaboration, inter-professional teamwork, and communication between various professionals in different healthcare organizations.

The aim of this paper is to identify challenges associated with collaboration in the daily healthcare work practice, among professionals in different healthcare organizations as well as the patients themselves and their relatives. A better understanding of the underlying challenges for collaboration can increase the potential to organize the collaborative work, and also to align IT systems with the collaborative work. The paper is structured as follows; a literature review is presented, followed by materials and methods. Then the thematized results are presented. The paper ends with a discussion and the conclusions drawn from the study.

## 2. Literature Review

Healthcare practices can be characterized as a socio-technical system of people and their skills, attitudes, beliefs and interactions, IT systems, techniques, organizational routines, activities, materials, documents and so on [6,7]. Within these practices, humans perform work supported by other factors, which results in interdependent patient care. The social and technical subsystems within the healthcare practices should be designed to optimize the performance of healthcare for frail elderly people [7]. IT systems are included in a larger system of activity within healthcare, and they are used in different ways for different professionals in order to perform work activities. IT systems should provide a seamless web created to support the work performed. People, organizations, and IT systems should not be viewed as separate domains with separate logics. Rather, they should be viewed as interrelated parts of a larger unit that represents a whole [6]. However, IT systems are still not fully integrated in the daily healthcare work practices [18].

Since professionals at different healthcare organizations largely perform independently from one another, their different perceptions and sensemaking structures need to be taken into account [19]. Even if identical IT systems are implemented at different departments within a healthcare organization, there is a chance that the work tasks develop differently within the different organizations [20]. IT systems often fail to meet healthcare professionals’ expectations and needs and are therefore not always considered optimal and efficient [21]. This, in turn, also means that difficulties emerge when using IT systems for collaborative work or for sharing of information and knowledge. Hence, healthcare professionals need better IT solutions [13].

Still, the world of healthcare is faced with the IT silo problem [14,22]. The IT silo problem is considered to be systemic and consists of difficulties in connecting and integrating a diverse ecology of systems, organizations, and users. Healthcare is provided by different healthcare organizations that need to collaborate to some degree. IT systems should support these different healthcare work practices with different information silos [23].

Since the problems are of a socio-technical nature, focusing merely on solving the technical aspects of the problems is not enough. Clearly, the implementation of new IT systems will challenge procedures and structures within healthcare [24]. However, in order to be useful, IT systems need to support the characteristics of the healthcare work practices performed in reality.

Inter-professional collaboration has to be based on teamwork and communication. To achieve effective teamwork, professionals in different healthcare organizations need to have a shared mental model. A shared mental model can be described as a common understanding of a situation, of a treatment plan, and of the roles and work tasks of the various professionals in the team. A shared mental model based on common information and knowledge facilitates adjusting strategies when needed and supports problem solving and decision making related to patient care [18]. IT systems need to be well integrated in the work activities in order to support the task being conducted in the team-based work where professionals in different healthcare organizations need to collaborate.

## 3. Materials and Method

### 3.1. Methodological Approach

This paper was based on a qualitative study conducted within an inter-organizational healthcare setting in the western part of Sweden. The study focused on collaboration between healthcare professionals from different healthcare organizations involved in healthcare for frail elderly people. Since the study had been geared towards a specific work practice, it was of great importance to use a methodological strategy that is adapted for research of the applied nature of the empirical setting [25]. Data collection was conducted through qualitative focus group interviews and concerned views and experiences expressed by people [26]. Thus, what types of actions performed within healthcare has been of interest in this study, as well as the work performed in the healthcare work practice.

### 3.2. Empirical Setting

Swedish healthcare has three different organizational levels. Firstly, there are the hospitals, which are responsible for the organization on a regional level. Secondly, there are the primary healthcare organizations, which have branches in every municipality assigned to certain responsibilities that differ from those of the hospitals. Thirdly, the municipalities themselves function as healthcare organizations, as they have personnel employed to care for frail elderly people in their homes.

Coordinated care is based on a value system that emphasizes three main issues of healthcare. The first main issue focuses on the protection of patients’ privacy, normality and right to self-determination, and person-centered healthcare. The second main issue highlights the obligations of the personnel groups to provide healthcare that is good and safe, both from a social and a medical perspective. Appropriate working methods and encouragement to interact and cooperate shall safeguard that the healthcare meets these criteria. The third issue focuses on the economic perspective, which prioritizes that patients in inpatient care should be rapidly discharged and that statutory objectives should be respected [27]. The core activities involved in healthcare work include investigation, monitoring and intervening of patients, as well as managing the patients’ trajectories, in order to cure them. All those tasks are to some extent performed collectively. Hence, there is a need for cooperation, collaboration, and negotiations between healthcare professionals in different healthcare organizations. The work is often of an ad hoc nature and performed in emergency situations where decisions have to be pragmatic. In this sense, healthcare work is complex and impossible to fully predict.

One of the main functions of the IT systems that are used within the coordinated healthcare process is to support statutory transfer of information in the form of messages that are sent between healthcare professionals when patients are admitted or readmitted to hospital. IT systems have functions for leaving messages about healthcare demands, admission information, calls and information in relation to forthcoming healthcare planning, decided healthcare plans, and information about readmission. IT systems also have functions of informational and administrative character. For patients in need of healthcare after their discharge from hospital, coordinated healthcare planning should be carried out in the hospital (SOSFS 2005:27). In such coordination processes that involve different healthcare organizations, IT systems should be used.

When a hospital physician decides to readmit a patient, the municipality social worker concerned should receive information about this from the hospital via an IT system. After that, the social worker should contact other professionals in the municipality as well as a nurse at the hospital in order to arrange a care planning meeting. The municipal home care nurse and primary healthcare professionals also receive messages via an IT system about the readmission of the patient to hospital. In the care planning process, it is necessary that representatives from all relevant organizations participate, and that those representatives have skills that enable them to meet the patient’s need for interventions after discharge. However, the responsibility for the patient’s care after discharge is not transferred from the hospital to the primary healthcare, the home care services, the social workers, or the municipal home care services to such an extent that would be favorable for the patient. Primary healthcare organizations often do not participate in the care planning process to a great extent, and a specific district physician who is to be responsible for the patient is often lacking.

### 3.3. Data Collection

The data were collected through three focus group interviews. By choosing to use focus groups, it was possible to maximize the exploration of different perspectives on healthcare for frail elderly people. The use of focus groups facilitated communication between the participants in each group, as they were able to express their views, ideas, and suggestions [28,29]. Each focus group consisted of eight professionals and one family member representative of the frail elderly people patient group. To increase the study’s validity, each professional participant was carefully selected in order to represent one type of profession from one specific healthcare organization. Family member representatives were included in order to obtain their perspectives to increase data validity. The focus group participants did not work together on a daily basis, but they represented collaborating organizations. Each focus group consisted of the following participants: One hospital physician, one general practitioner physician, one hospital nurse, one primary healthcare nurse or district nurse, one community nurse, one municipal assistance officer or social worker, one rehabilitation staff member working for either the primary healthcare services or for the municipality (a physiotherapist or an occupational therapist), as well as one family member representative of frail elderly patients. Two researchers were present at each focus group interview. One of them functioned as a moderator to facilitate the group discussion, whereas the other one either functioned as an observer, or assisted the moderator by asking follow-up questions.

Three sets of vignettes, one for each focus group, were constructed in the form of typical cases to be used in the interviews. Each vignette described the situation of a frail elderly patient in need of coordinated healthcare from several different healthcare organizations. Before being used in the focus group interviews, the vignettes were also validated by a group of healthcare professionals. Each focus group interview lasted for two hours. The topics raised during the interviews were hindrances and opportunities for coordinated efforts aiming to decrease the need for readmission of frail elderly people. The interviews also focused on finding common and constructive solutions for increased collaboration between different healthcare organizations that would be possible to implement in reality. The focus group interviews were tape-recorded, and were transcribed verbatim afterwards.

### 3.4. Data Analysis

The analysis of the collected data focused on interpreting the participants’ communication and the dynamics within each group. As human thought processes are complex, the interpretive method was focused on the complexity of human sensemaking within the focus groups, and on how the communication and dynamics evolved [30]. The analyzed data set consisted of the three transcribed focus group interviews. Thematic analysis was used for identifying and analyzing themes within the data set [31]. Each theme was defined based on whether it captured something important in relation to the overall aim of this paper, as the analysis was driven by this particular aim. Since the focus group interviews were concerned with an overall perspective on the situation and collaboration in healthcare for frail elderly patients, this thematic analysis was conducted in order to provide a more detailed and nuanced account on particular themes in relation to the use of IT systems. Moreover, the themes were identified based on an inductive approach, in which the themes are strongly linked to the data set. Thus, the coding process was conducted without allowing any analytic preconceptions [31].

Another important aspect of this research was concerned with the latent, or interpretative, level of analysis. That means that the themes were identified not only based on what was explicitly expressed in the data, but also based on underlying ideas, assumptions, and conceptualizations that could be perceived [29]. This can be exemplified by the participants’ talking about problems encountered when using IT systems without them mentioning those IT systems explicitly in the interviews. This kind of latent content analysis was possible thanks to the researchers’ experience and knowledge from previous empirical studies in the context of IT systems use in healthcare. By considering both latent and manifest content in the data analysis, the thematic analysis becomes more nuanced [32]. Content detected in the data is thus seen as an expression of latent content. In this way, latent data content is inseparable from the manifest content. The analysis was inspired by a constructionist perspective, since it was assumed that meanings and experiences are socially constructed. Therefore, the perspective was directed towards theorizing structural conditions within the inter-organizational context.

## 4. Study Results

The study identified challenges experienced by different professionals and family member representatives within the context of healthcare for frail elderly patients. The challenges were related to the use of IT systems for communication and sharing of information and knowledge in the daily and collaborative work practice of healthcare professionals. The identified challenges were structured into four different themes based on the thematic analysis. These themes were: (1) Insufficient information exchange, (2) inconsistencies in communication, (3) differences in the use of IT systems, and (4) deficient coordination.

### 4.1. Insufficient Information Exchange

Professionals in different healthcare organizations, relatives, as well as the patients themselves are not always aware of all the information that exists about the patients. Many of them state that they do not know what has been said, and that they do not receive any information. One municipal assistance officer expressed:

“There are many patients and even relatives who say they do not really know what has been said. They find that they have not really received information… and many feel frustrated when they come home because they do not know… they do not even know who will follow up and so…”

When a patient is about to be discharged from a hospital, nurses at hospital departments register information in an IT system. Hospital professionals assess the patient’s need for interventions and support at home after discharge; however, physicians do not participate in the care planning activities. If interventions and support at home is considered necessary, a care planning meeting has to be arranged. After approval from the patient, this information is registered in an IT system. This IT-system is separate from the electronic patient records, as medical information is not included in the same data set. The separate information sets are subject to different laws and can therefore not be merged. All healthcare organizations can access and add information in this IT system, but many professionals claim that the information in the IT system is insufficient. A municipal assistance officer stated:

“I wish that there was even more information about just the need for support at home, the experience of feeling safe when coming home and … when we are calling… when we talk to the Healthcare Planning Unit then they enter the IT system and they can read what is written. I know that nurses have a great deal of work to do, but it would be desirable to have even more information. If we know more about the patient’s history social situation and feelings of safety, we can cooperate even better.”

Hospital nurses are responsible for arranging healthcare planning meetings and for documenting agreements between separate healthcare organizations regarding what interventions and support the patient needs, when it should be provided and by which organization, as well as what equipment the patient needs to be able to stay at home. Many nurses, however, consider the IT system insufficient in handling the amount of information needed for documenting the patient’s healthcare needs. The municipal social workers, on their part, need information about what kind of interventions and support the patient needs at home, which they often claim is missing in the IT system. Consequently, the municipal social workers repeatedly need to contact the hospital nurses to obtain this information.

Simultaneously, primary care professionals can also access the information provided by the hospital. However, while primary care nurses frequently log into the IT system to check patients’ information provided by the hospitals, primary care physicians seldom access this information. Even though physicians have the right to access this IT system, and the information it contains, they often consider it too time consuming to retrieve information from the IT system. Hence, they typically delegate this monitoring work to the primary care nurses. In addition, hospital physicians seldom pass on information to their colleagues in the primary care services. The primary care services are therefore less well-informed, and hence less involved, in the healthcare of frail elderly patients when they arrive home after being discharged from hospital.

### 4.2. Inconsistencies in Communication

The information provided by the IT system is not always coherent. A patient’s different professional contacts at different healthcare organizations often have questions regarding the latest news about the patients and their overall status. The professionals expressed a desire to inform their colleagues at other collaborating healthcare organizations, but they claimed that the IT system does not fully support this sharing of information. Work routines as well do not support communication between healthcare organizations. This was expressed by a primary healthcare nurse:

“The IT system KLARA is where we see patients coming in and out of the hospital and whether they have home care or not home care, or whether there should be any care planning or similar, so that, yes, I think we work a lot separately, always actually.”

In order to divide the various healthcare responsibilities for a patient, hospitals and primary healthcare organizations should improve their communication. Communication should be established between the hospital and the primary care services in order to reach an agreement on responsibility for the patient. The current routines, however, often cause long delays. Communication delays of about three months are not uncommon, and by the time a hospital physician receives confirmation from the primary care services regarding their responsibility for a patient, it is very likely that the hospital physician has forgotten about the patient in question. Relatives also notice the lack of communication, expressed as follows: “*After all, it’s a lack of communication, absolutely. As a relative, I see it almost daily, then, that it is just a lack of communication*.” There is no efficient system for communicating transfers of responsibility between separate healthcare organizations. The work routines do not support the communication between professionals in different healthcare organizations either, as one general practitioner physician expressed:

“It is extremely rare that hospital colleagues call me, but without it sometimes sick patients are moved from hospital to the municipal short-term accommodation on Thursday afternoon or Friday morning. Then they are not actualized in the IT system until Tuesday and some of these patients are at the end of life.”

New manual systems that involve sending new specific fax forms back and forth are currently being introduced, but many physicians are not yet aware of these forms and their related routines. A hospital physician expressed it at follows:

“We use preliminary epicrises and faxes, but we never make calls. I think there was a new form developed, but I am not sure if it has been fully introduced.”

### 4.3. Differences in the Use of IT Systems

Different departments have different ways of using the IT system for collaboration between healthcare organizations. At some healthcare organizations the system is used for care planning, and a physio therapist as well as an occupational therapist are consulted before a patient’s care plan is entered into the IT system. Hence, the IT system seems to be difficult to use sometimes for the coordination of healthcare, as a hospital nurse expressed: “*The IT system KLARA, it’s a little complicated, I think all my nurse colleagues think so too*.”

At other departments, the IT system is only used for minimal documentation of information. As a consequence, primary care professionals and municipal professionals receive different kinds of information from different hospital departments. A hospital physician expressed the problem as follows:

“Communication is written, as epicrises and referrals, in the IT systems. We seldom talk directly to each other. Since documentation is lacking, it is not certain that medications are documented at all, nor how the professional think or make their decisions. It’s the other way too, that you request journal copies from the district and they say nothing to you. Documentation is very important.”

The use of IT systems in relation to management of medicines is problematic. There is no coordinated medicine list for the patient, as hospital physicians use a different system for prescribing drugs than primary care physicians. Both IT systems are used at pharmacies, and this might confuse patients as the hospital medicine list does not correspond with the list at the pharmacies. The separated systems also cause problems when a patient is readmitted to hospital, as hospital physicians cannot access information about any medicine previously ordinated by primary care physicians. Instead, they have to rely on information provided by the patients or their relatives, for example by bringing the medicine list when the patient is readmitted. A family member representative described the situation as follows:

“When it comes to IT support and medicine lists and all that…then you should not ask my mother, as a patient, how it is with this and that medicine right now. With 15 medications, how to keep track of them, I couldn’t do it! Now I do not know how it looks like in the IT system, but I know there are things to improve. You should not have to ask these questions to the patient, the information should be there in the IT system, for the physician.”

An additional problem is that if the medicine list originates from primary care services, it only contains medicines prescribed within the last year. Although there is an IT system for coordinating prescriptions between pharmacies, hospitals, primary care services, and municipal care services, it is not always used.

Electronic patient records are subject to a similar problem. Hospitals and primary care services use different types of electronic patient records for each patient, and municipal professionals do not have access to these records from all hospitals and primary care organizations. One district nurse expressed the necessity of one common IT system for patient records as follows: “*It should have been the same record system for everyone!*”

### 4.4. Deficient Coordination

Coordination between different healthcare organizations is not so developed, and is not supported by IT systems to such an extent. A district nurse believed that the situation has deteriorated:

“After all, nobody really has, any responsibilities nowadays, and there is no one the patient can call to speak, if you say so. In the past there were more district nursing departments, where we had different responsibilities. Now all that is gone, you could say, but before you always had someone to call, that is, someone you could turn to who knew the patient, who had a little bit of control, and someone who could help to sort things out”.

If healthcare professionals wish to, they can send excerpts from the electronic patient records to the other involved healthcare organizations. One primary care physician mentioned that during a period of six months, he had sent all the excerpts from his documentation in the electronic patient records to the hospital as soon as one of his patients was readmitted to hospital. However, the physician never received any response from the hospital regarding the information he had sent. The physician stated that he had never received any requests for information from the primary care services either. The hospital physician claimed that information about the patients’ medicines is requested from the secretaries. Thus, the work of physicians positioned in different healthcare organizations is almost never coordinated. Moreover, the current IT system does not support this kind of coordination. One primary healthcare nurse described the lack of coordination:

“The primary care physician care for this patient, but I would just like you to know that there are many who do not know who does what, and who to contact … but at the primary healthcare center we are not directly connected in this case, except from our physician who is responsible for home care, and as a nurse, I just see the patient in the IT system KLARA, and that is all…”

Furthermore, there is no IT system that can support coordination between separate healthcare organizations with regard to information used for motivating deprescription of medicines. Physicians at separate healthcare organizations do not have information about other physicians’ decisions regarding prescriptions or deprescriptions. One physician suggested that a reorganization of healthcare as a whole could contribute to solving coordination problems:

“My dream scenario is to merge the primary and municipality healthcare, to one and the same organization. Then I think it had worked much better. Now it is like, yes, this is mine and this is yours… If we are under the same roof, the responsibility would be different, I believe, and with better coordination, working together.”

When a patient is discharged from hospital, an epicrisis is written and given to the patient in the form of a paper copy. Primary care professionals and municipal professionals alike are authorized to read this epicrisis, but this requires that the patient presents it for them. Social workers also have access to the epicrises via the healthcare planning IT system. However, the epicrises do not contain information about what needs to be done in order to facilitate collaboration and coordination between separate healthcare organizations. The current IT systems, routines, and work activities do not support inter-professional and inter-organizational sharing of information and knowledge. Professionals in different healthcare organizations only contact each other in an ad hoc-based manner. As one community nurse expressed:

“The medicine handling can be very wrong in my experience. It is very messy and the patient may have medications at home, which they say they take too, so I would usually have to contact the home care physician, about how we should do, or sometimes I call the hospital and talk to the physician there.”

The concept of coordinated individual planning aims to support inter-organizational sharing of information and knowledge and is currently being introduced. Healthcare professionals can take on the responsibility for such coordinated planning efforts, but few healthcare professionals are aware of this opportunity. Relatives also recognize the lack of coordination. One family member representative claimed:

“What I miss all the time is that coordinator in the network of healthcare organizations who takes care of the patient. That person does not exist, or I have not been able to find that person who I think could solve so many of these gaps that we are talking about. If it is a fax that has to be sent or whatever it is, this person actually makes sure that it happens, someone who cares about the patient, and who has experience perhaps, preferably from many of these areas.”

However, not many healthcare professionals are aware of the coordinated planning opportunity. Furthermore, relatives recognize the deficiencies of coordination, as a family member representative expressed: “*What I react to here is precisely this because there is inadequate coordination and the patient himself feels confused and does not know what is going on around him*.”

## 5. Discussion

Within healthcare for frail elderly people, professionals in healthcare organizations need to communicate, cooperate, and coordinate their activities. While it is acknowledged that activities need to be documented, there seems to be some confusion regarding what to document. Safe and effective patient healthcare requires that the documentation contains information that professionals in other healthcare organizations need about the patient and about the patient’s medication and previously made decisions. One way to better understand what information other professionals need and improve the communication is to create teams of professionals around the patients [17].

At the same time, patients and their relatives should be considered as partners in healthcare decisions that concern them. In order to achieve an efficient organization of a patient’s healthcare, IT systems could be a means for supporting this communication and cooperation. Professionals in separate healthcare organizations face problems when trying to communicate and collaborate with each other, partly because IT systems are considered as fragmented and complex to use. Thus, IT systems are integrated in a distributed environment in complex and only partially defined activities and work processes [23]; that is, the work practices of the healthcare organizations are not defined in an appropriate way, and IT systems do not support effective communication and coordination.

IT systems can be referred to as IT silo systems, and Bygstad et al. claim that we need clear reasons for building them [13]. This study showed that procedures and structures within the different healthcare organizations can also be considered as silos. It is obvious that professionals in different healthcare organizations document what they consider necessary from their own perspective, while professionals in other healthcare organizations may need other information about a patient. Silo problems in healthcare practices become problematic when healthcare professionals are not coordinating and communicating based on the patient’s interests and safety. Rather than focusing on who might be the receiver of the information documented, the documenting professional focuses on his or her own needs. Attitudes and behavior seem to be based neither on coordination and communication, nor on common information that is used by all healthcare professionals. Hence, the challenge consists of building IT systems that can support both coordination and sharing of information and knowledge. There is a lack of a shared mental model regarding the understanding of the patient’s situation, the plan for treatment, and the roles and tasks of the professionals in charge in different healthcare organizations [17].

A number of different IT systems are used within the healthcare work practice. To some extent, IT systems consist of an infrastructure that generates and transfers information and knowledge about patients. However, professionals in the different healthcare organizations face problems when they communicate and collaborate with each other, partly since IT systems are considered as fragmented and complex to use. However, as long as healthcare organizations lack common perspectives and sufficient understanding of how collaboration should be conducted, IT systems alone cannot be blamed for the shortcomings. Current IT systems are not capable of matching the professionals’ different expectations and needs, according to Blegind Jensen and Aanestad [19]. This study finds that healthcare organizations do not deliver safe patient care of good quality. The IT systems lack functions to fully support communication and collaboration [17], though it is difficult to change work routines and processes without having prerequisite for inter-professional teamwork and communication [21]. Each healthcare organization has different opportunities to selectively adopt and use the capabilities of IT systems. In other cases, politicians as well as managers at the regional organizational level select which capabilities IT systems should provide within each healthcare practice. IT systems are not perceived as designed for supporting actual and necessary collaboration. However, even if IT systems support communication and collaboration, they have not been designed to change work practice and its routines. Thus, it is common to have an over-reliance on IT systems, assuming they should contribute to creating changes in work practice. Then, one could blame IT systems for posing hindrances for sharing of information and knowledge. Offering healthcare for frail elderly people becomes increasingly complex. IT systems are being developed to support collaboration and communication between different healthcare organizations. Nonetheless, without developing the work processes and routines, reaching a common understanding regarding information and knowledge sharing, and establishing appropriate coordination among professionals in the different healthcare organizations IT systems alone cannot be expected to solve the existing problems.

Within healthcare, many activities do not result in the creation of records. Berg states that record-creating activities should be more transparent and controllable, and that they are of importance for the inter-professional relations within healthcare [7]. Collaboration as well as knowledge sharing activities do not usually result in creation of records neither on paper, nor electronically. In fact, even though there is a need for professionals to collaborate both within a healthcare organization and between different healthcare organizations, such collaboration is not emphasized by IT systems as they are not used for creating records or other pieces of information. Organizations are not ready for appropriate work processes for collaboration. IT systems are not supporting collaborative, inter-professional, and inter-organizational work in order to optimize health care for frail elderly people [8].

Further research should be conducted on the collaboration and coordination of healthcare between different healthcare organizations, in order to find solutions on how to further develop healthcare work practices and provide safe healthcare of good quality for frail elderly patients, as well as how IT systems can be developed to support this.

## 6. Conclusions

The study identified challenges associated with the use of IT systems within the following themes: Insufficient information exchange, inconsistencies in communication, differences in the use of IT systems, as well as deficient coordination. Professionals in healthcare organizations, patients, and relatives do not have access to all the information that they need. Communication between different healthcare organizations is problematic due to unclear division of responsibilities. Different IT systems are used in different ways, and there are deficiencies in the coordination among them. Hence, the exchange of information and coordination of activities do not fully support the provision of healthcare for frail elderly people. Several professionals from different healthcare organizations are involved in the healthcare for frail elderly people, and many of them do not have a full understanding of the patients’ needs and the full range of roles and work tasks that healthcare organizations need to provide. The professional work performed in different healthcare organizations, as well as the IT systems used, can be considered as based in silos. In order to better understand how IT systems could support collaboration between professionals from different healthcare organizations, work processes and routines should be based on a common understanding and on inter-professional teamwork within the healthcare services. The work processes that aims to promote collaboration between different healthcare organizations need to be better organized, and the use of IT systems needs to be better aligned.
